# Comprehensive analysis identifies CLEC1B as a potential prognostic biomarker in hepatocellular carcinoma

**DOI:** 10.1186/s12935-023-02939-1

**Published:** 2023-06-12

**Authors:** Qiangan Jing, Chen Yuan, Chaoting Zhou, Weidong Jin, Aiwei Wang, Yanfang Wu, Wenzhong Shang, Guibing Zhang, Xia Ke, Jing Du, Yanchun Li, Fangchun Shao

**Affiliations:** 1 Laboratory Medicine Center, Department of Clinical Laboratory, Zhejiang Provincial People’s Hospital, Affiliated People’s Hospital, Hangzhou Medical College, Hangzhou, Zhejiang China; 2 Department of Central Laboratory, Affiliated Hangzhou first people’s Hospital, Zhejiang University School of Medicine, Hangzhou, Zhejiang China; 3 College of Biotechnology and Bioengineering, Zhejiang University of Technology, Hangzhou, Zhejiang China; 4 Department of Hematology, The first people’s Hospital of Fuyang Hangzhou, Hangzhou, Zhejiang China; 5 Cancer Center, Department of Pulmonary and Critical Care Medicine, Zhejiang Provincial People’s Hospital, Affiliated People’s Hospital, Hangzhou Medical College, Hangzhou, Zhejiang China

**Keywords:** CLEC1B, Hepatocellular carcinoma, Tumor microenvironment, Immunoregulator, Sorafenib, Biomarker

## Abstract

**Background:**

C-type lectin domain family 1 member B (CLEC1B, encoding the CLEC-2 protein), a member of the C-type lectin superfamily, is a type II transmembrane receptor involved in platelet activation, angiogenesis, and immune and inflammatory responses. However, data regarding its function and clinical prognostic value in hepatocellular carcinoma (HCC) remain scarce.

**Methods:**

The expression of CLEC1B was explored using The Cancer Genome Atlas (TCGA) and Gene Expression Omnibus (GEO) databases. RT-qPCR, western blot, and immunohistochemistry assays were employed to validate the downregulation of CLEC1B. Univariate Cox regression and survival analyses were used to evaluate the prognostic value of CLEC1B. Gene Set Enrichment Analysis (GSEA) was conducted to investigate the potential association between cancer hallmarks and CLEC1B expression. The TISIDB database was applied to search for the correlation between immune cell infiltration levels and CLEC1B expression. The association between CLEC1B and immunomodulators was conducted by Spearman correlation analysis based on the Sangerbox platform. Annexin V-FITC/PI apoptosis kit was used for the detection of cell apoptosis.

**Results:**

The expression of CLEC1B was low in various tumors and exhibited a promising clinical prognostic value for HCC patients. The expression level of CLEC1B was tightly associated with the infiltration of various immune cells in the HCC tumor microenvironment (TME) and positively correlated with a bulk of immunomodulators. In addition, CLEC1B and its related genes or interacting proteins are implicated in multiple immune-related processes and signaling pathways. Moreover, overexpression of CLEC1B significantly influenced the treatment effects of sorafenib on HCC cells.

**Conclusions:**

Our results reveal that CLEC1B could serve as a potential prognostic biomarker and may be a novel immunoregulator for HCC. However, its function in immune regulation should be further explored.

**Supplementary Information:**

The online version contains supplementary material available at 10.1186/s12935-023-02939-1.

## Introduction

Hepatocellular carcinoma (HCC), characterized by high incidence and mortality rates [1], is a major public health problem that causes severe disease and economic burdens on humans, according to global statistical data [2, 3]. It is projected that by 2025, the morbidity rate of HCC will exceed one million events annually [4]. HCC is the most common type of liver cancer, accounting for 90% of all primary liver cancers. Although the chance of cure for early-stage HCC can be increased by local ablation, surgical resection, and liver transplantation, most HCC patients are generally diagnosed at an advanced stage because early-stage HCC has no obvious symptoms [3]. Other non-negligible factors including rapid proliferation, invasion, and metastasis lead to traditional radiotherapy and chemotherapy being limited and ineffective for HCC [5, 6]. Therefore, it is imperative to identify new therapeutic targets for HCC.

Previous studies confirmed that immune checkpoint blockers (ICBs), used as the main method of immunotherapy, have markedly conferred survival benefits for some advanced tumors, including bladder cancer, clear cell renal cell carcinoma, melanoma, and others [7–10]. A growing body of literature supports the notion that the tumor microenvironment (TME) is an essential part of the tumor and serves as a complicated ecosystem that plays an important role in tumor initiation, metastasis, and resistance to immunotherapy [11, 12]. For example, the immune response can be reflected by the determined TME context of diagnosis [7], and the prognosis of patients is closely associated with changes in the infiltration of CD8^+^ T cells, CD4^+^ T cells, and macrophages in the TME. Zhu [13] and colleagues revealed that the clinical benefit was tightly associated with pre-existing immunity (CD274 expression, T–effector signature, and intratumoral CD8^+^ T cells density) in patients with HCC when treated with atezolizumab (anti-PD-L1) combined with bevacizumab. Although immunotherapy is an effective approach for advanced patients to achieve a therapeutic effect, only a small proportion of patients respond to and benefit from it because of the primary or secondary resistance mechanisms to ICB in the complex TME [14, 15]. To increase the response rate of immunotherapy, exploring a novel biomarker or immunomodulator that correlates with the TME is essential.

CLEC1B belongs to the Dectin-1 gene cluster and encodes C-type lectin-like receptor 2 (CLEC2), with a molecular weight of approximately 32 KDa [16]. Previous studies have shown that CLEC1B is not only expressed in platelets but also in immune cells (e.g., myeloid cells, dendritic cells, macrophages, and NK cells) and is involved in tumorigenesis, development, and metastasis [17]. It regulates various signaling pathways by recognizing and binding to its ligands. In colon cancer, CLEC1B has been shown to suppress tumor metastasis and platelet aggregation [18]. Data have shown that CLEC1B is significantly downregulated in HCC and that the proliferation and migration of HCC cells can be inhibited by the overexpression of CLEC1B [19, 20]. In recent years, an increasing number of reports have revealed the crucial role of CLEC1B in immune and inflammatory responses. Hu et al. demonstrated that the low and high expression of CLEC1B and PD-L1, respectively, may be valuable prognostic markers associated with the response to ICB therapies [21]. Rayes et al. showed that podoplanin-CLEC-2 can be a new anti-inflammatory axis that regulates the infiltration of immune cells [22]. However, the potential prognostic value of CLEC1B and its effect on immune-related TME components in HCC has not been completely elucidated.

In this study, we evaluated the abnormal expression of CLEC1B in HCC and normal hepatic tissues and further validated the downregulation of CLEC1B transcriptional and protein expression. In addition, we conducted prognostic value, immune infiltration, immunological correlation, and enrichment analyses of CLEC1B. We found that the expression of CLEC1B significantly affects the clinical survival outcomes of HCC and may play an immunologic enhancement role in the HCC tumor microenvironment. Importantly, we confirmed that the expression of CLEC1B affects the cytotoxicity of sorafenib on HCC cells. In conclusion, our results revealed that CLEC1B could be a promising biomarker for the treatment and prognostic assessment of HCC.

## Materials and methods

### Expression analysis of CLEC1B

The RNA-sequencing data of 33 types of tumors and adjacent normal tissues were obtained from the UCSC XENA database (https://xenabrowser.net/datapages/). The level 3 HTSeq-FPKM data and LIHC patient clinical data were derived from the TCGA database (https://portal.gdc.cancer.gov/). The gene expression data (fragments per kilobase million, FPKM), which contained 374 tumor samples and 50 adjacent normal events, was first transformed to transcripts per kilobase million (TPM) format, and then TPM was transformed to log2 for subsequent analysis. In parallel, four data sets GSE121248, GSE76427, GSE36376, and GSE60502 that related to HCC were retrieved from GEO datasets (https://www.ncbi.nlm.nih.gov/geo/). When a gene with multiple expression values, the mean value was taken for its expression level. “ggplot2” R package was performed for the visualization of expression analysis, and Wilcoxon rank sum test and Wilcoxon signed-rank test were used for the assessment of significance. The Human Protein Atlas database (HPA: https://www.proteinatlas.org/) and the GeneCards database (https://www.genecards.org/) were used to profile the expression of CLEC1B in normal liver tissue. The HCCDB database (http://lifeome.net/database/hccdb/home.html) and the Oncomine database were performed to confirm the expression of CLEC1B in HCC.

### Survival analysis

The GEPIA2 database (http://gepia2.cancer-pku.cn/#index) was performed to analyze the overall survival (OS) and disease-free survival (DFS). The “rms” and “survival” R packages were used for constructing the Cox proportional hazards regression model, and drawing the Kaplan-Meier survival curves, nomogram, and calibration curve of the nomogram. The “maxstat” R package was implied for the calculation of the best cut-off values and Log-rank tests were used for the assessment of significance.

### CLEC1B expression in immune cells

The “RNA immune cell” module of the Human Protein Atlas database was used to assess the CLEC1B expression in immune cells. In addition, the Tumor Immune Single-cell Hub (TISCH) database (http://tisch.comp-genomics.org/home/) was applied for single-cell analysis [23].

### Immune-related analysis

LIHC patients were divided into CLEC1B-high and CLEC1B-low groups according to the median value of CLEC1B expression. The correlation between CLEC1B expression and infiltrating immune cells was explored by using the TISIDB database (http://cis.hku.hk/TISIDB/) [24]. Markers for 24 distinct immune cells and their classification has been described in the study of Bindea et al. [25]. The sample gene-set enrichment analysis (ssGSEA) algorithm was conducted to calculate the abundance of immune cells and represented by enrichment scores [26–28]. The “estimate” R package was used for the calculation of the stromal score, immune score, and estimated score in the individual patient. The “maxstat” R package was carried out to calculate the optimal cutoff values of the stromal score, immune score, and estimated score, and then patients were divided into high-and low score groups. We then used the survfit function of the “survival” R package to analyze the prognostic difference between the high-and low score groups, and Log-rank tests were used for assessing the significance. In addition, the multivariable Cox proportional hazard model based on immune cell infiltration and CLEC1B expression was explored using the TIMER database (http://timer.cistrome.org/). The correlations between CLEC1B and 122 immunomodulators were calculated by using the Sangerbox tool (http://vip.sangerbox.com/login.html) [29, 30].

### Enrichment analysis based on CLEC1B co-expressed genes

The “h.all.v7.2.symbols.gmt”, “c5.all.v7.2.symbols.gmt”, and “c2.cp.kegg.v7.2.symbols.gmt” gene sets were downloaded from the Molecular Signatures Database (MSigDB) (https://www.gsea-msigdb.org/gsea/index.jsp) [31]. The “clusterProfiler” was utilized for GSEA analysis with the following parameters [32]: false discovery rate (FDR) < 0.05, number of permutations = 1000. Visualization of GSEA was depicted by the “ggplot2” R package under the conditions of |normalized enrichment score (NES)| > 1 and *P*-adjusted value < 0.05. The immune subtype of LIHC patients based on CLEC1B expression was analyzed by using the TISIDB database. The “DESeq2” R package was used to screen the differentially expressed genes (DEGs) that are associated with CLEC1B. Genes with *P*-adjusted value < 0.05, and |log2FoldChange| > 1 were considered to be DEGs. Gene ontology (GO) and Kyoto Encyclopedia of Genes and Genomes (KEGG) analysis was performed using the “clusterProfiler” and “org.Hs.eg.db” R packages, the “ggplot2” R package was utilized for visualization.

### PPI network construction and enrichment analysis of CLEC1B-interacted proteins

20 proteins that interacted with CLEC1B were obtained from the STRING database (https://string-db.org/) and the PPI network was visualized by Cytoscape software. Function annotation, including the biological process and KEGG pathway analysis, was conducted by the ClueGO software, which can extract representative functional information of these genes [33].

### Cell culture, plasmids, and western blot

Liver cancer cell lines (SMMC-7721, Huh7, MHCC-97 H, and HCC-LM3), normal hepatic cell line (LO2), and 293T cells were preserved in our laboratory and cultivated in DMEM (Hyclone) supplemented with 10% (v/v) fetal bovine serum (Gibco), 100 U/ml penicillin and 0.1 mg/ml streptomycin. Cells were grown in an incubator of 5% CO_2_ at 37 ℃. The pCMV3-Flag-CLEC1B plasmids were designed and confirmed by the Sino Biological (Beijing, China). The DYKDDDDK Tag (9A3) Mouse mAb (cat# 5750s, Cell Signaling Technology, China), CLEC1B Rabbit pAb (cat#A9971, ABclonal, China), and β-Actin rabbit mAb (cat# AC026, ABclonal, China) were applied for western blot analysis, and the dilution rate of the antibodies were 1:2,000, 1:1,000, and 1:5,000, respectively. Image J software was used for the quantification of western blot bands.

### Tissue microarray (TMA) and immunohistochemistry

The human HCC TMA was obtained from a commercialized company (http://www.cancercell.com.cn/). Immunohistochemical detection of CLEC1B expression was performed according to our previously published literature [34]. Briefly, the TMA slides were placed in an oven at 65 °C for 2 h, dewaxing and dehydration with xylene and graded ethanol, respectively. After antigen retrieval, 1% bovine serum albumin was used to block the nonspecific binding. Then, the slides were incubated with anti–CLEC1B rabbit polyclonal antibody (cat#DF14376, Affinity Biosciences, AUS) overnight at 4 ℃. After washing with PBS for 3 times, the slide was incubated with biotin–labeled secondary antibodies. Finally, slides were visualized by the DAB staining kit, and the Nikon DS-Ri2 microscope (Japan) was applied for image capture. The Image J software was used for the quantization of protein expression levels.

### Cell viability assay

SMMC-7721 plvx-neo and SMMC-7721 CLEC1B-neo (8,000 cells/well) were seeded in 96 (NEST Biotechnology) well plates and administrated with sorafenib (5, 10 µM) for 24 h after cell attachment. Subsequently, added CCK8 reagent (10 µL/well) and incubated for 1 h at 37 ℃. Ultimately, the microplate reader was performed to measure the absorbance at 450 nm.

### RNA isolation and RT-qPCR

Total mRNA was extracted by SteadyPure RNA Extraction Kit (Accurate Biotechnology, China) based on the manufacturer’s instructions. Quantification of mRNA was carried out by NanoDrop One (Thermo Scientific). Reverse transcription was conducted via Evo M-MLV RT Mix Kit (Accurate Biology, China) according to the manufacturer’s instructions. The quantitative PCR was performed utilizing SYBR Green Premix Pro Taq HS qPCR Kit (Accurate Biology, China) reagents according to the manufacturer’s recommendations, and the β-actin housekeeping gene was used as the endogenous reference. Primers of CLEC1B and GAPDH were indicated below: CLEC1B forward, 5’−GCTGCTATGGGTTCTTCAGG-3’, reverse, 5’−TCCCACTTCCAGACCTCATT-3’; GAPDH forward, 5’−GCACCGTCAAGGCTGAGAAC-3’, reverse, 5’−ATGGTGGTGAAGACGCCAGT-3’. The 2^−ΔΔCt^ method was used to analyze the relative mRNA expression levels of target genes.

### Cell apoptosis assay

Cells (6 × 10^5^/well) were plated in 6 well plates and treated with sorafenib (10 µM) for 24 h after cell attachment. Then, cells were digested with pancreatin and washed with PBS. After that, Annexin V-FITC/PI apoptosis kit (Multi-Science, China) was used for the detection of cell apoptosis according to our previously published literature [35, 36]. Finally, flow cytometry was used for determining the apoptosis distribution.

### Statistical analyses

The Wilcoxon signed-rank test and Wilcoxon rank-sum test were performed to assess the significance of CLEC1B expression in paired or non-paired samples, respectively. We used the Univariate Cox regression analysis and Kaplan-Meier approach to evaluate the prognostic value of CLEC1B. For survival curves, the Log-rank test was performed to estimate the difference. The Spearman correlation analysis was carried out to assess the statistical significance between CLEC1B and other factors. The *t*-test and one-way ANOVA was used to evaluate the significance of two and multiple groups comparison, respectively. All statistical analyses were performed with R 4.1.0 software or GraphPad Prism 9.0, and *P* < 0.05 was considered significant.

## Results

### The expression analysis and tumor suppression role of CLEC1B

**Fig. 1:**
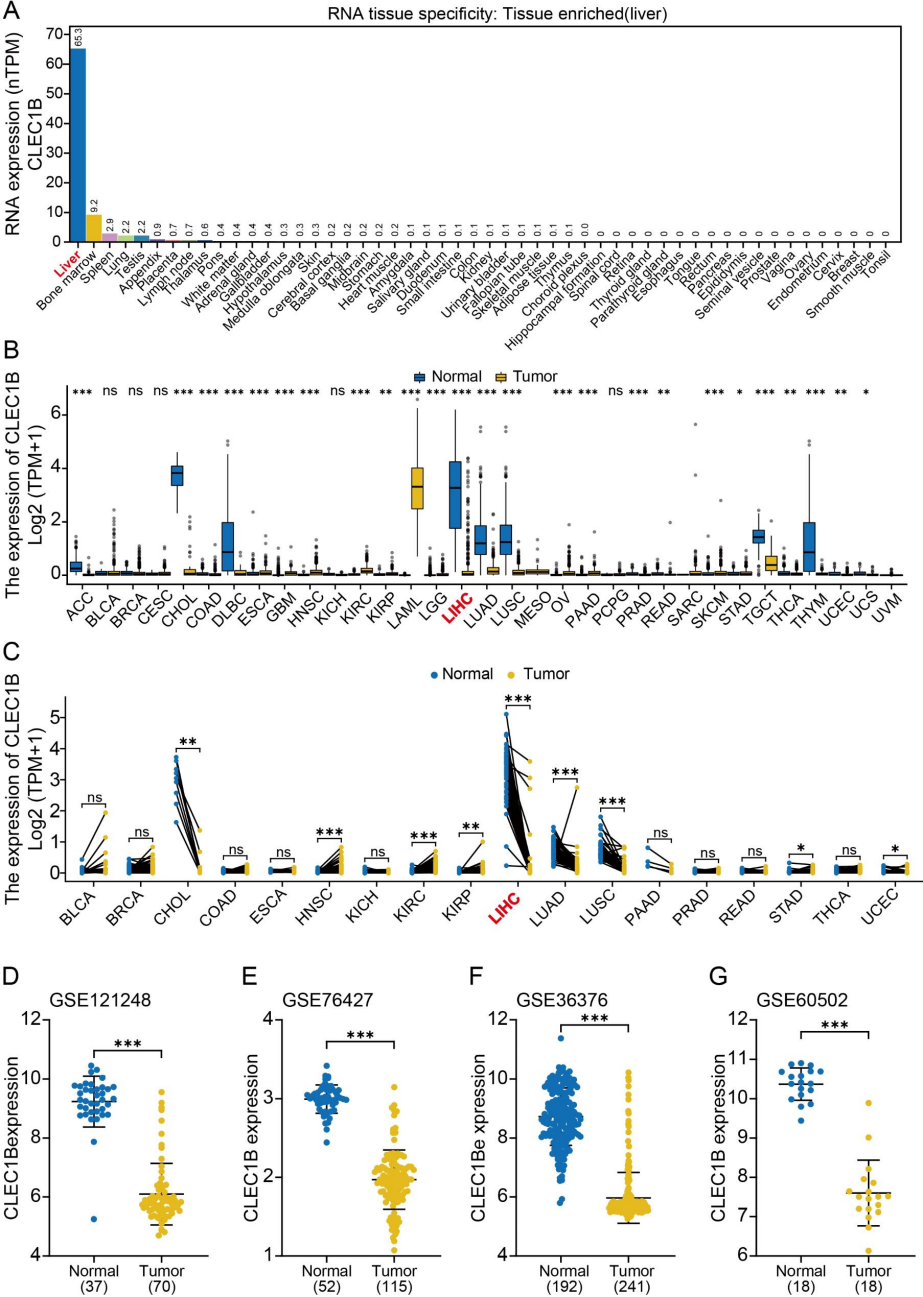
Expression profile of CLEC1B in HCC. (**A**) RNA expression of CLEC1B in normal tissues based on the HPA database. (**B**) Box plot of CLEC1B expression in 33 cancer types. (**C**) Transcriptional expression of CLEC1B in paired samples of 18 cancer types. (**D**-**G**) Scatter diagrams of CLEC1B expression in HCC according to the GEO database. *P-value < 0.05, **P-value < 0.01, ***P-value < 0.001

We aimed to explore the potential role of CLEC1B and understand whether its expression affects tumorigenesis in HCC. Results showed that CLEC1B was mainly located in the plasma membrane and was highly expressed in the liver (Fig. 1A, Figure S1A, B). The analysis of the protein concentration of CLEC1B in the human plasma revealed a concentration of 1.5 µg/L (Figure S1C). We reasoned that this might be due to a physiological leakage of intracellular CLEC1B protein, suggesting that CLEC1B may could act as a biomarker.

We next analyzed the expression of CLEC1B in tumors and adjacent normal tissues across multiple cancer types. We observed that the mRNA expression of CLEC1B in LIHC, cholangiocarcinoma (CHOL), and lymphoid neoplasm diffuse large B-cell lymphoma (DLBC), was lower than that in the adjacent normal tissues (Fig. 1B, Figure S1D). In paired tumor tissues and adjacent normal tissues, the expression of CLEC1B also significantly decreased in various tumors, including LIHC (Fig. 1C, Figure S1E). Receiver operating characteristic (ROC) analysis revealed that the CLEC1B mRNA expression in LIHC was 0.986 (95% CI: 0.976–0.996) and the best cut-off value of CLEC1B was 1.783 TPM (Figure S1F). By analyzing four GEO datasets (Table S1), we discovered that the mRNA levels of CLEC1B in HCC were dramatically lower than those in normal hepatic tissues (Fig. 1D-G). Reports from the Chen Liver, Roessler Liver, Roessler Liver 2, and Wurmbach Liver in the Oncomine database also validated the low expression of CLEC1B in HCC (Figure S2A-D). Results from the HCCDB database confirmed the inferior transcription levels of CLEC1B in HCC (Figure S2E, F). Collectively, these data demonstrate that the expression of CLEC1B might play a tumor suppression role in the liver.

### The low expression of CLEC1B is correlated with poor prognosis in HCC patients

**Fig. 2:**
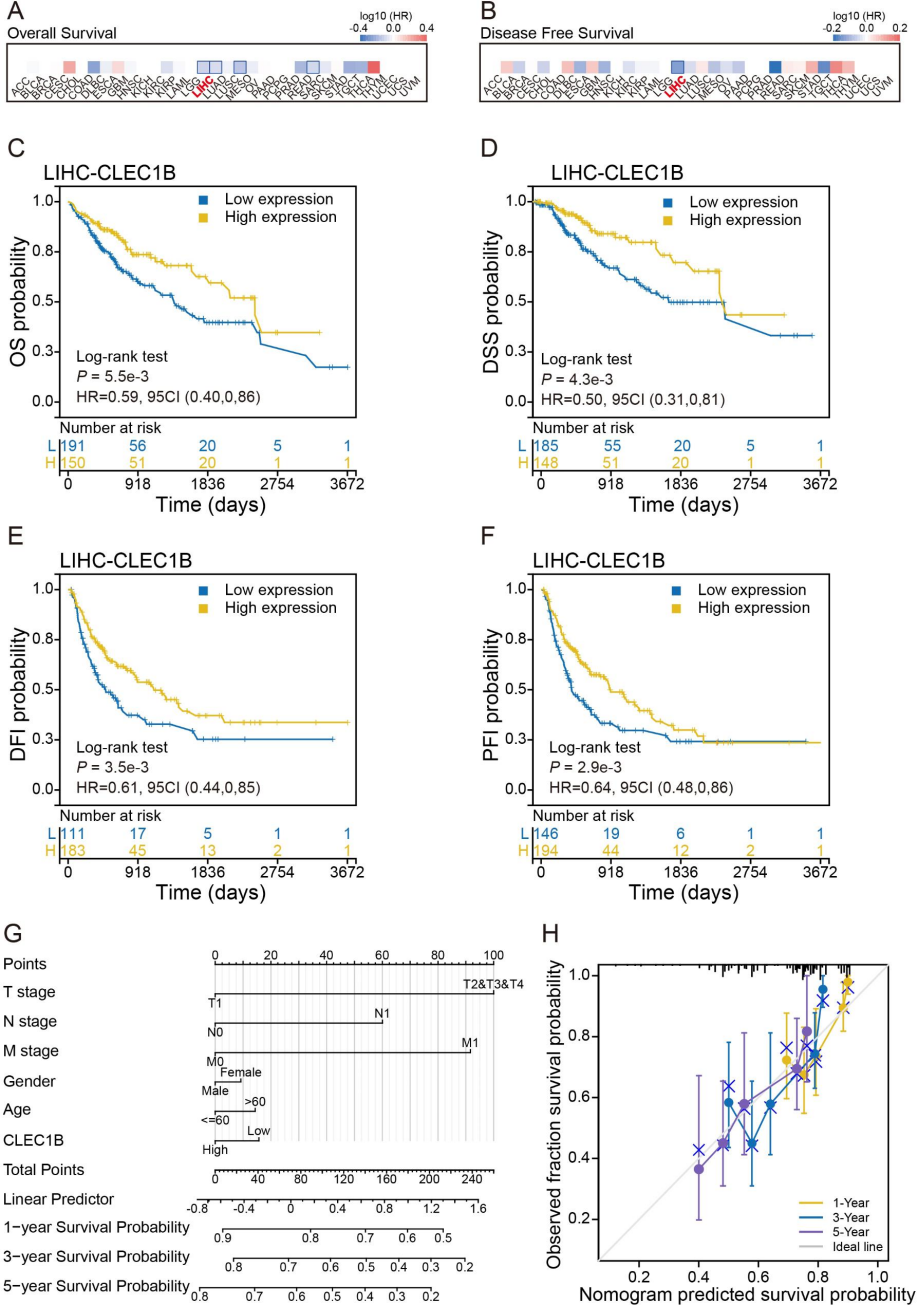
Prognostic significance of CLEC1B in HCC patients. (**A**, **B**) Heatmap showing the impact of CLEC1B on OS (**A**) and DFS (**B**). (**C**-**F**) The survival curves of OS (**C**), DSS (**D**), DFI (**E**), and PFI (**F**) from the TCGA data. (**G**) The nomogram of prognostic factors of LIHC, including CLEC1B expression and other clinical parameters according to TCGA data. (**H**) The calibration curve shows the nomogram’s performance of CLEC1B.

The association between CLEC1B expression and clinical characteristics was identified to further explore the role of CLEC1B in HCC. Results showed that CLEC1B was closely linked with the survival outcome of patients with HCC (Figure S3A-C). Univariate Cox regression analysis suggested that the mRNA expression of CLEC1B is an independent prognostic factor of overall survival (OS), disease-specific survival (DSS), disease-free interval (DFI), and progression-free interval (PFI) for HCC (Figure S3D). Similar results were obtained from the GEPIA2 database (Fig. 2A, B). Based on the profile of CLEC1B expression, survival analysis displayed that HCC patients with high CLEC1B expression had favorable survival outcomes (Fig. 2C-F). To verify whether CLEC1B can act as an independent prognostic factor for HCC, we constructed a model for the prediction of OS by incorporating CLEC1B expression and other clinicopathological information (Fig. 2G). The calibration curve, which was used to assess the nomogram’s performance for CLEC1B, showed the predicted survival probability, which provided an appreciable C-index of OS of 0.648 (Fig. 2H).

### CLEC1B expression level in various immune cells

**Fig. 3:**
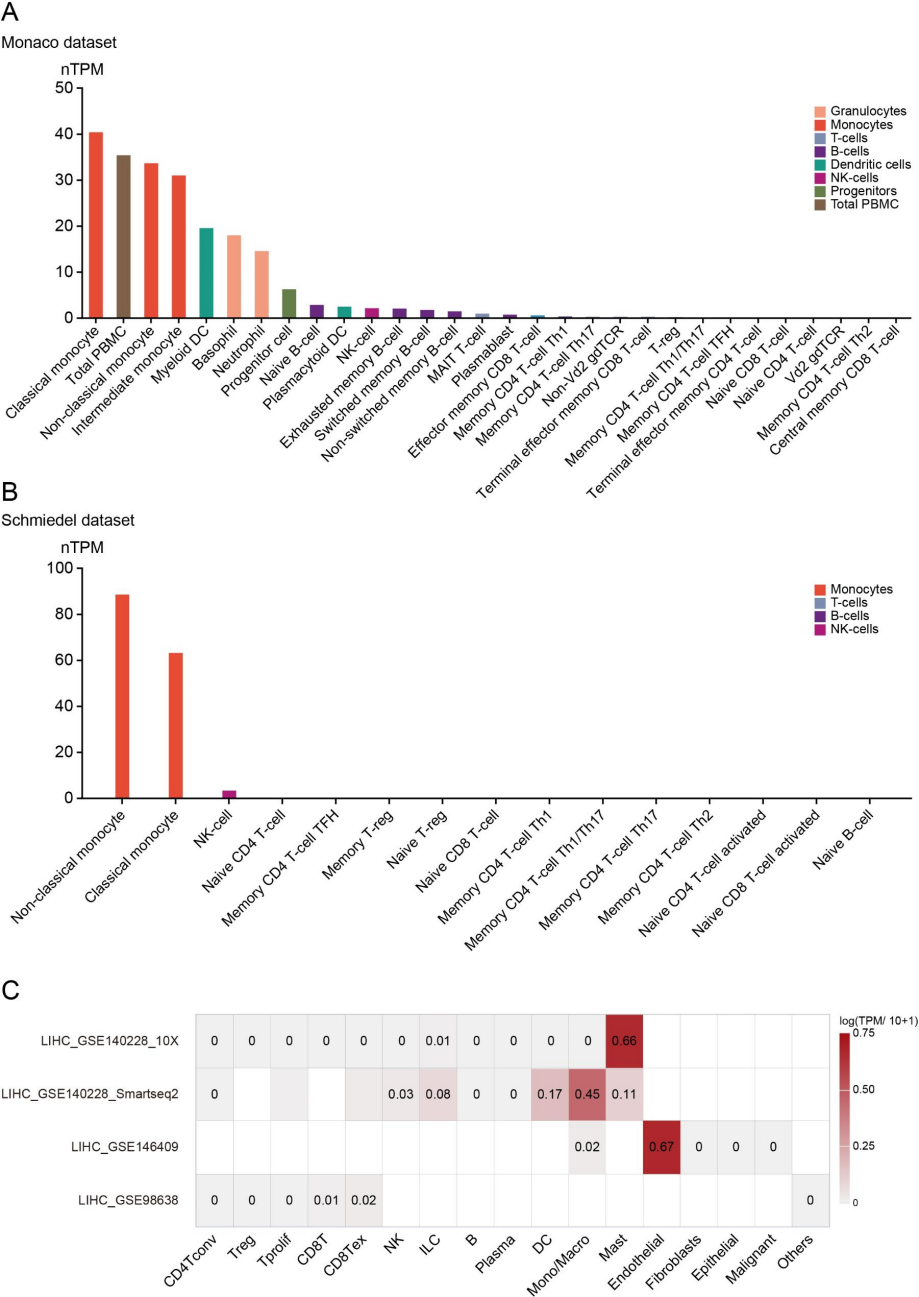
Expression of CLEC1B in immune cells. (**A**, **B**) The expression level of CLEC1B in various immune cells is based on the Monaco dataset (**A**) and Schmiedel dataset (**B**). (**C**) Summary of CLEC1B expression at the single cell level in four HCC datasets by the TISCH database

To gain a clear insight into whether CLEC1B expression in the immune cell was specific, its expression in immune infiltrating cells in plasma was explored by using the HPA database. CLEC1B was mainly expressed in monocytes, peripheral blood mononuclear cell (PBMC), myeloid dendritic cells, basophils, as well as neutrophils in the Monaco dataset (Fig. 3A). In the Schmiedel dataset, CLEC1B was primarily expressed in monocytes (Fig. 3B). Based on the online tool TISCH, single-cell analysis was performed to further investigate the expression of CLEC1B at the single cell level. Results showed that CLEC1B was predominantly expressed in endothelial cells, mast cells, and monocytes/macrophages in HCC (Fig. 3C).

### The expression of CLEC1B is closely correlated with immune infiltration

**Fig. 4:**
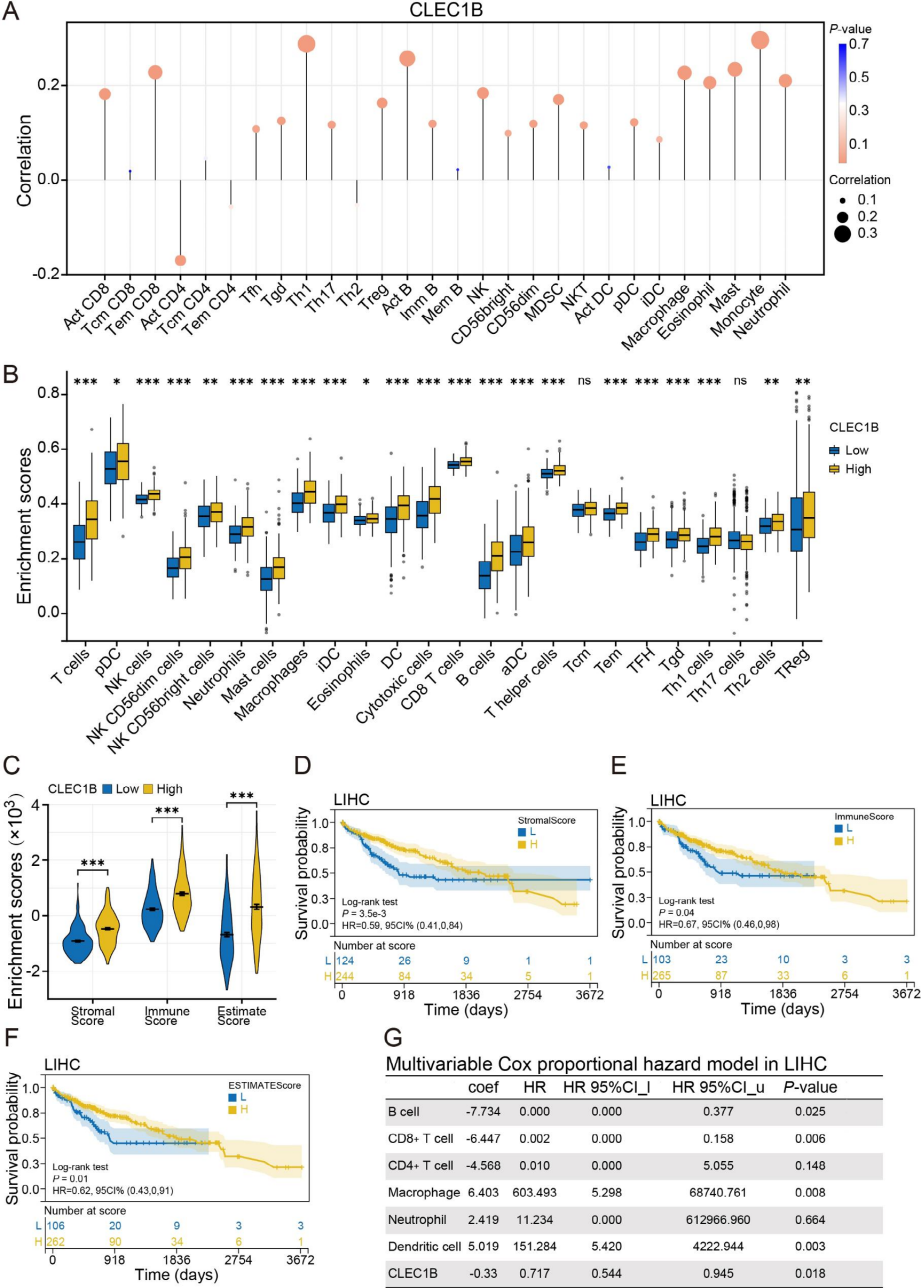
Immune infiltration analysis. (**A**) Correlation analysis of CLEC1B expression and various infiltrating immune cells. (**B**) Immune infiltration levels of 24 types of immune cells in the CLEC1B-high and − low groups. (**C**) The difference of stromal score, immune score, and estimated score in CLEC1B-high and − low groups. (**D**-**F**) Survival curves show the prognostic difference between the high-and low-score groups. (**G**) Multivariable Cox proportional hazard model according to infiltration of 6 types of immune cells and CLEC1B expression. *P-value < 0.05, **P-value < 0.01, ***P-value < 0.001

The physiological state of the TME, which consists of epithelial cells, vascular and lymphatic vessels, cytokines, chemokines, and infiltrating immune cells, is highly associated with tumor development and metastasis [37, 38]. To confirm the essential role of CLEC1B in the immune landscape of HCC, we first explored the correlation between CLEC1B expression and multiple immunological markers. Results illustrated in Fig. 4A and Table S2 indicated that CLEC1B was positively correlated with the infiltration levels of most tumor-infiltrating lymphocytes, including monocyte, Th1, Act B, Tem CD8, Act CD8, macrophage, and others. GSVA was performed to explore the distinct infiltration levels of 24 types of immune cells in CLEC1B high-and low-expression groups. Interestingly, the immune infiltration density of most TME cells was higher in CLEC1B high expression group than in the CLEC1B low-expression group (Fig. 4B).

It is now well appreciated that stromal and immune cells are the two main types of non-neoplastic components, their infiltration levels greatly affect tumor earlier diagnosis and prognostic evaluation [39]. Immune and stromal scores were commonly utilized to predict the infiltration of non-tumor cells by analyzing specific gene expression characteristics of immune and stromal cells [40], and the estimate score is a composite score of immune and stromal scores used for tumor purity evaluation. The “ESTIMATE” algorithm proved that the CLEC1B high expression group boasts higher stromal scores, immune scores, and estimate scores (Fig. 4C). In parallel, HCC patients with elevated stromal scores, immune scores, or estimate scores conferred a favorable prognosis in contrast with the CLEC1B low expression population (Fig. 4D-F). Furthermore, multivariable Cox proportional hazard model findings revealed that infiltration of CD8^+^ cells, B cells, macrophages, and dendritic cells may influence the prognosis of HCC (Fig. 4G). Altogether, the role of CLEC1B in the tumor microenvironment of HCC may be intricate, but it is compelling to focus on its effect on immune cell infiltration.

### Immunological correlation and GSEA of CLEC1B

**Fig. 5:**
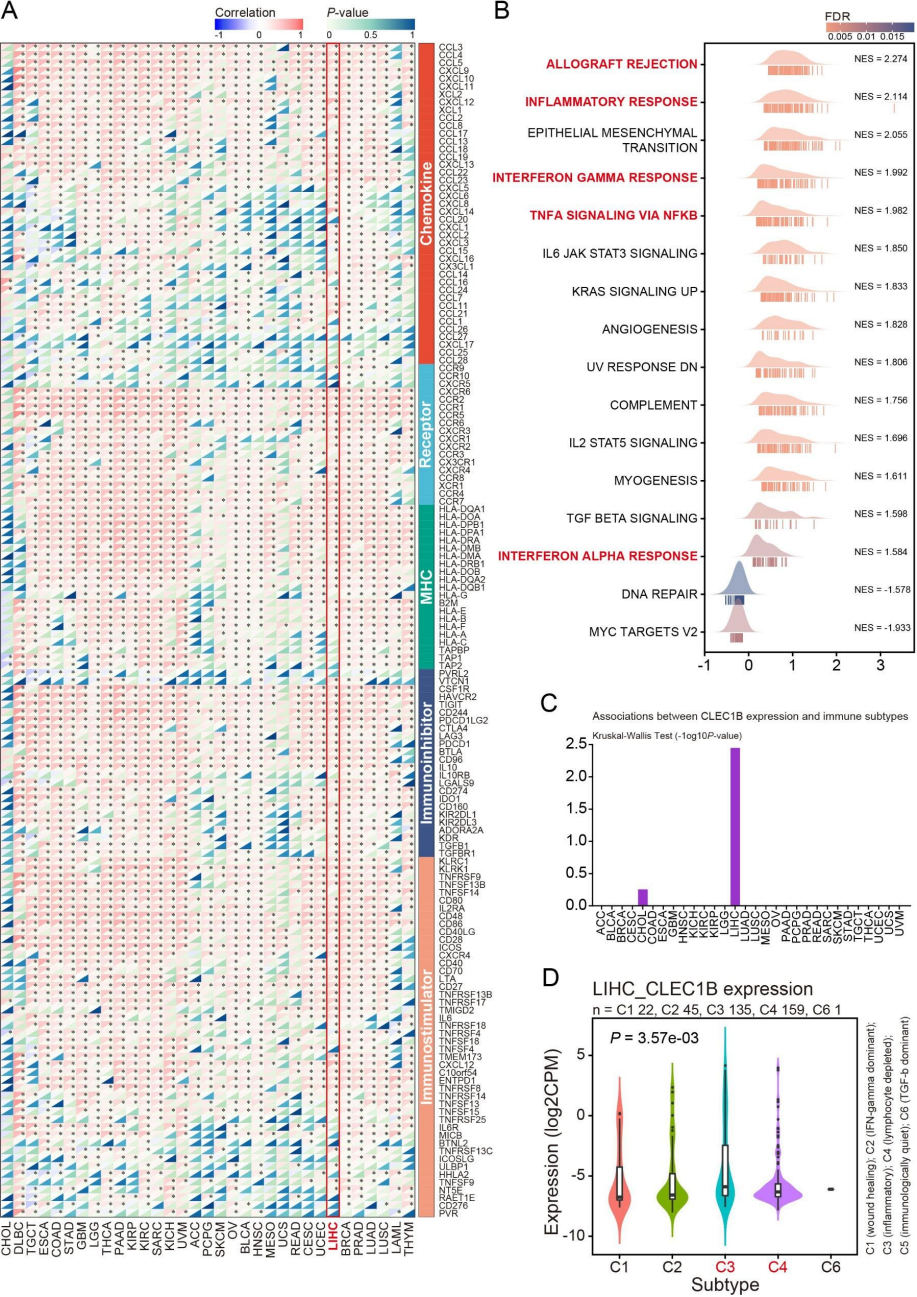
Immunological correlation and GSEA of CLEC1B. (**A**) Correlation analysis between CLEC1B expression and 122 types of immunomodulators. (**B**) GSEA of CLEC1B based on the hallmarks gene set. (**C**, **D**) Association between CLEC1B expression and immune subtypes. *P-value < 0.05

To further depict the immunological role of CLEC1B, correlation analysis was conducted and revealed that CLEC1B was positively associated with the bulk of immunomodulators (chemokines, receptors, major histocompatibility complexes, immunoinhibitors, and immunostimulators) in HCC (Fig. 5A). GSEA was performed to further discern the CLEC1B-related cancer hallmarks, and we found that CLEC1B expression was significantly associated with immune-related pathways, including “allograft rejection”, “inflammatory response”, “interferon gamma response”, “TNFA signaling via NFKB”, and “interferon alpha response” (Fig. 5B). Moreover, the epithelial mesenchymal transition (EMT) pathway was markedly related to CLEC1B expression. Accumulating evidence suggests that the EMT is strongly linked to tumorigenesis, metastasis, and drug resistance [41, 42], implying that CLEC1B may play an essential role in tumor onset and development by engaging in the EMT. Furthermore, the expression of CLEC1B was associated with the immune subtypes of HCC patients, and different expression level of CLEC1B was observed among the five immune subtypes (C1: wound healing; C2: IFN-γ dominant; C3: inflammatory; C4: lymphocyte depleted; C6: TGF-β dominant), with the highest and lowest expression in C3 and C4 subtypes, respectively (Fig. 5C, D). Studies unveiled that the C3 immune subtype exhibits a type I immune response and the most pronounced Th17 signature, which has the best prognosis and may represent immune equilibrium, whereas the C4 subtype confers the worst survival outcome, is dominated by macrophages and displays low lymphocytic infiltration [43]. In summary, these data indicate that CLEC1B expression is capable of affecting the immune activation status of HCC.

### Function annotation of CLEC1B based on its co-expressed genes

**Fig. 6:**
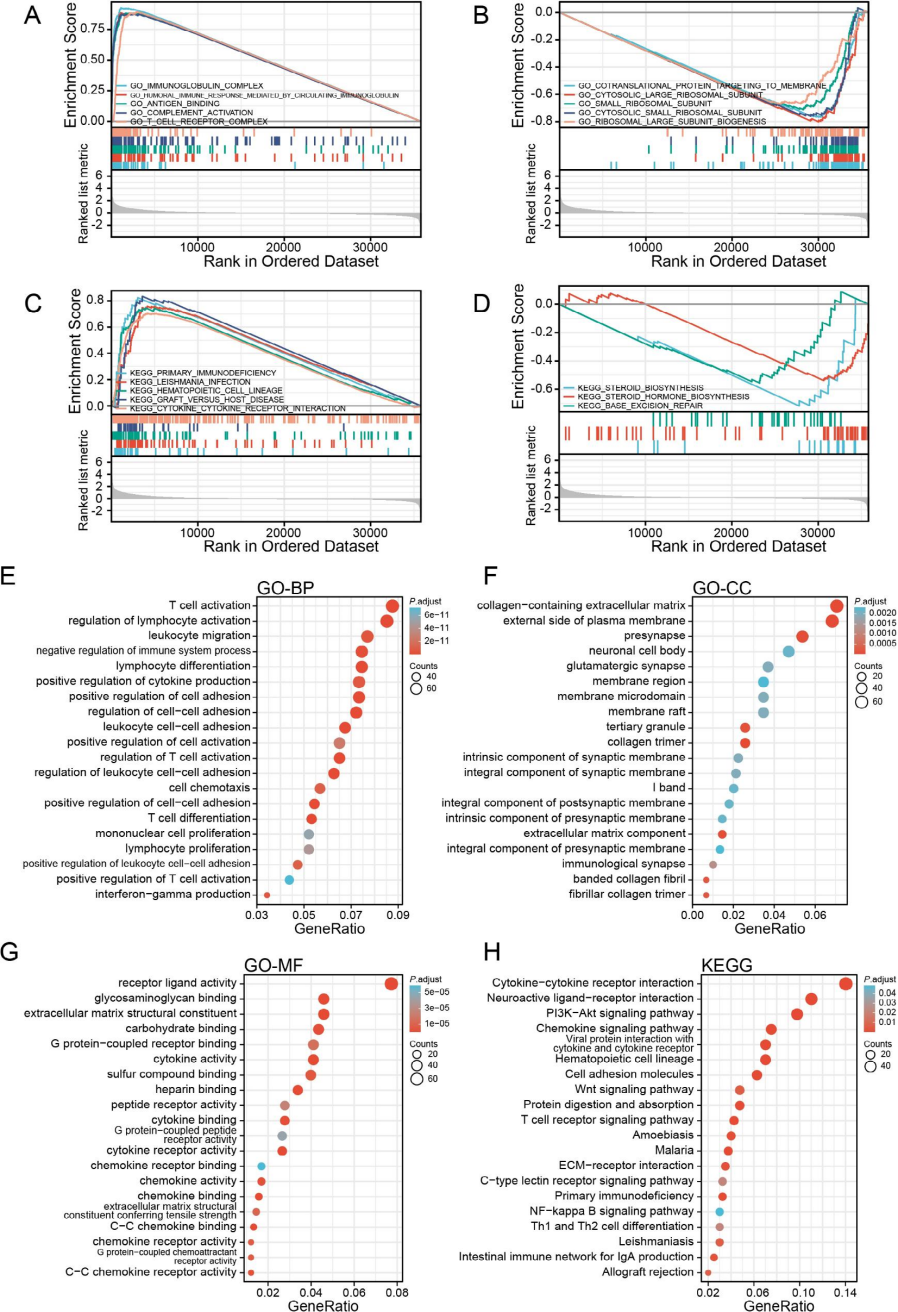
Functional enrichment analysis of CLEC1B co-expression genes in HCC. (**A**, **B**) GO terms analysis revealed the most five positively correlated pathways (**A**) and the five most negatively correlated pathways (**B**). (**C**, **D**) KEGG pathways unveiled the most five positively correlated pathways (**C**) and the three most negatively correlated pathways (D). (E-H) BP (**E**), CC (**F**), MF (**G**), and KEGG pathways (**H**) enrichment analysis according to CLEC1B and its co-expression genes, dot size stands for the number of genes, and the color of the dot represents the adjust *P*-value

We next execute GSEA to discern the CLEC1B-associated biological processes (BP) and signaling pathways. Results showed that the top five GO items that most positively related to the expression of CLEC1B were “immunoglobulin complex”, “humoral immune response mediated by circulating immunoglobin”, “antigen binding”, “complement activation”, and “T cell receptor complex all associated with immune regulation” (Fig. 6A). Whereas, the top five GO items that are most negatively related to CLEC1B expression seem to be related to the ribosome (Fig. 6B). In addition, CLEC1B expression was significantly associated with “primary immunodeficiency”, “leishmania infection”, “hematopoietic cell lineage”, “graft versus host disease”, and “cytokine-cytokine receptor interaction” signaling pathways (Fig. 6C). While, negatively correlative with “steroid biosynthesis”, “steroid hormone biosynthesis”, and “base excision repair” signaling pathways (Fig. 6D).

Furthermore, 946 DEGs that co-expressed with CLEC1B were identified and used to GO and KEGG enrichment analyses to explore the functional mechanism of CLEC1B in HCC development. We discovered these DEGs were predominantly enriched in immune-related BP (“T cell regulation”, “regulation of lymphocyte activation”, “negative regulation of immune system processes”, “lymphocyte differentiation”, “positive regulation of cytokine production”, etc.) (Fig. 6E). These genes were principally enriched in the cellular components (CC) terms “collagen-containing extracellular matrix”, “external side of the plasma membrane”, “presynapse neuronal cell body”, and “glutamatergic synapse” (Fig. 6F), the molecular function (MF) terms “receptor ligand activity”, “glycosaminoglycan binding”, “extracellular matrix structural constituent”, “carbohydrate binding”, as well as “G protein-coupled receptor binding” (Fig. 6G). KEGG analysis proved that they were implicated in “cytokine-cytokine receptor interaction”, “neuroactive ligand-receptor interaction”, “PI3K-Akt signaling pathway”, “chemokine signaling pathway”, and “viral protein interaction with cytokine and cytokine receptor” (Fig. 6H). These observations indicate that CLEC1B and its co-expressed genes may likely participate in immune regulation.

### PPI network construction and enrichment analysis of CLEC1B-interacted proteins

**Fig. 7:**
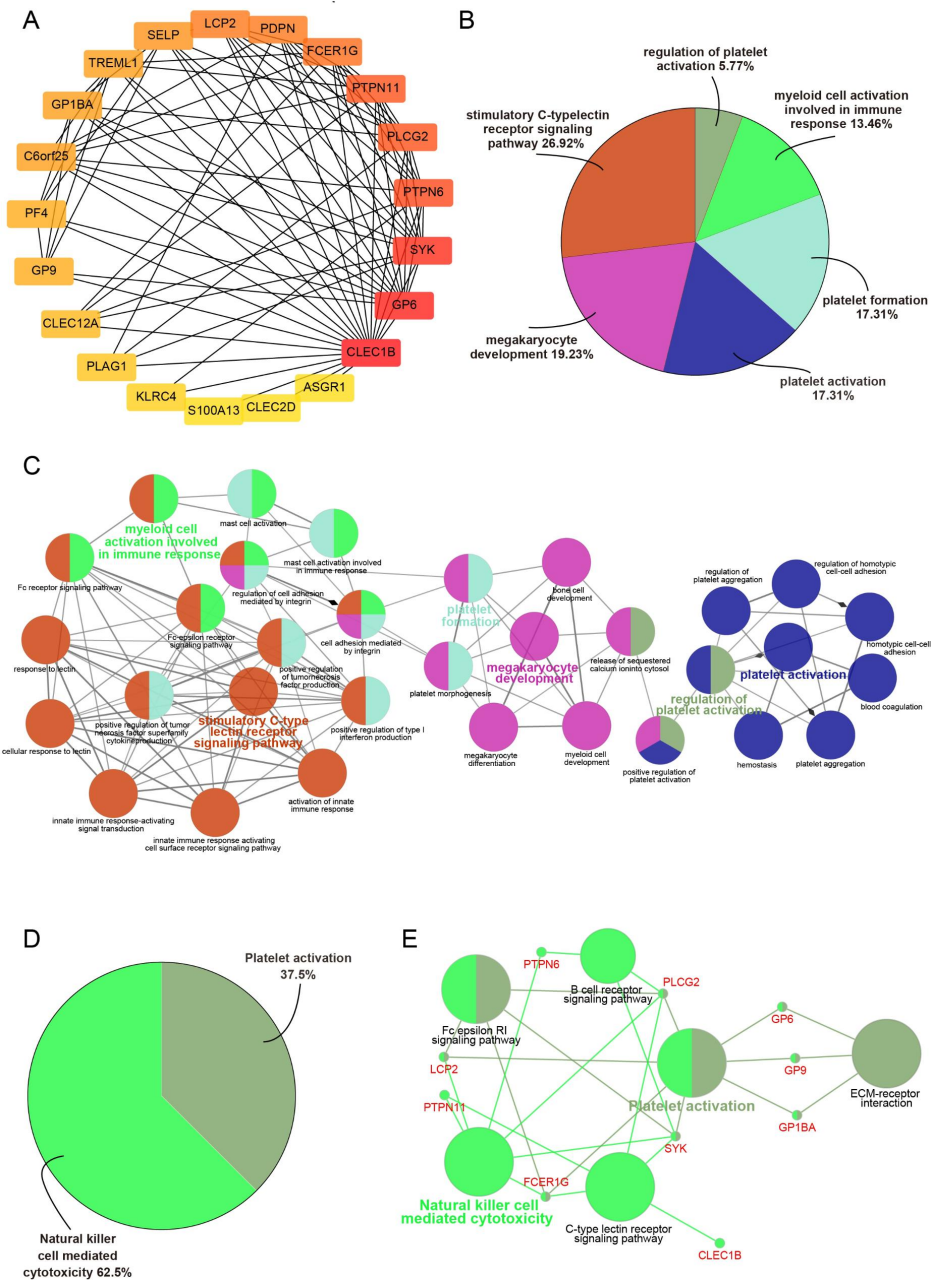
Identification of CLEC1B interacting proteins and function annotation. (**A**) PPI network of 20 interaction proteins with CLEC1B. (**B**) The pie chart showed the percentage of 6 GO items. (**C**) GO analysis of the 21 proteins visualized by ClueGO. (**D**) The pie chart showed the distribution proportion of 2 KEGG pathways. (**E**) KEGG pathway analysis of these proteins

To further illustrate the molecular mechanisms of the signaling pathways mediated by CLEC1B-related partners in HCC, 20 proteins that interacted with CLEC1B were obtained from the STRING database, and the PPI network was visualized using Cytoscape (Fig. 7A). We found that the mRNA expression levels of 15 of the 20 proteins were positively correlated with CLEC1B expression, and the top five genes of relevance were CLEC12A, SELP, LCP2, SYK, and GP1BA (Figure S4A, Table S3). The GO terms revealed that these genes were enriched for 32 GO items and were involved in multiple biological processes, including “regulation of platelet activation”, “platelet formation”, “megakaryocyte development”, “myeloid cell activation involved in immune responses”, and “stimulatory C-type lectin receptor signaling pathway” (Fig. 7B, C). The KEGG pathway analysis indicated that they mostly participated in “natural killer cell-mediated cytotoxicity”, “B cell receptor signaling pathway”, “Fc epsilon RI signaling pathway”, and “C-type lectin receptor signaling pathway” (Fig. 7D, E). The above results suggest that CLEC1B may be an essential immunomodulatory factor in HCC.

### The expression validation of CLEC1B in HCC

**Fig. 8:**
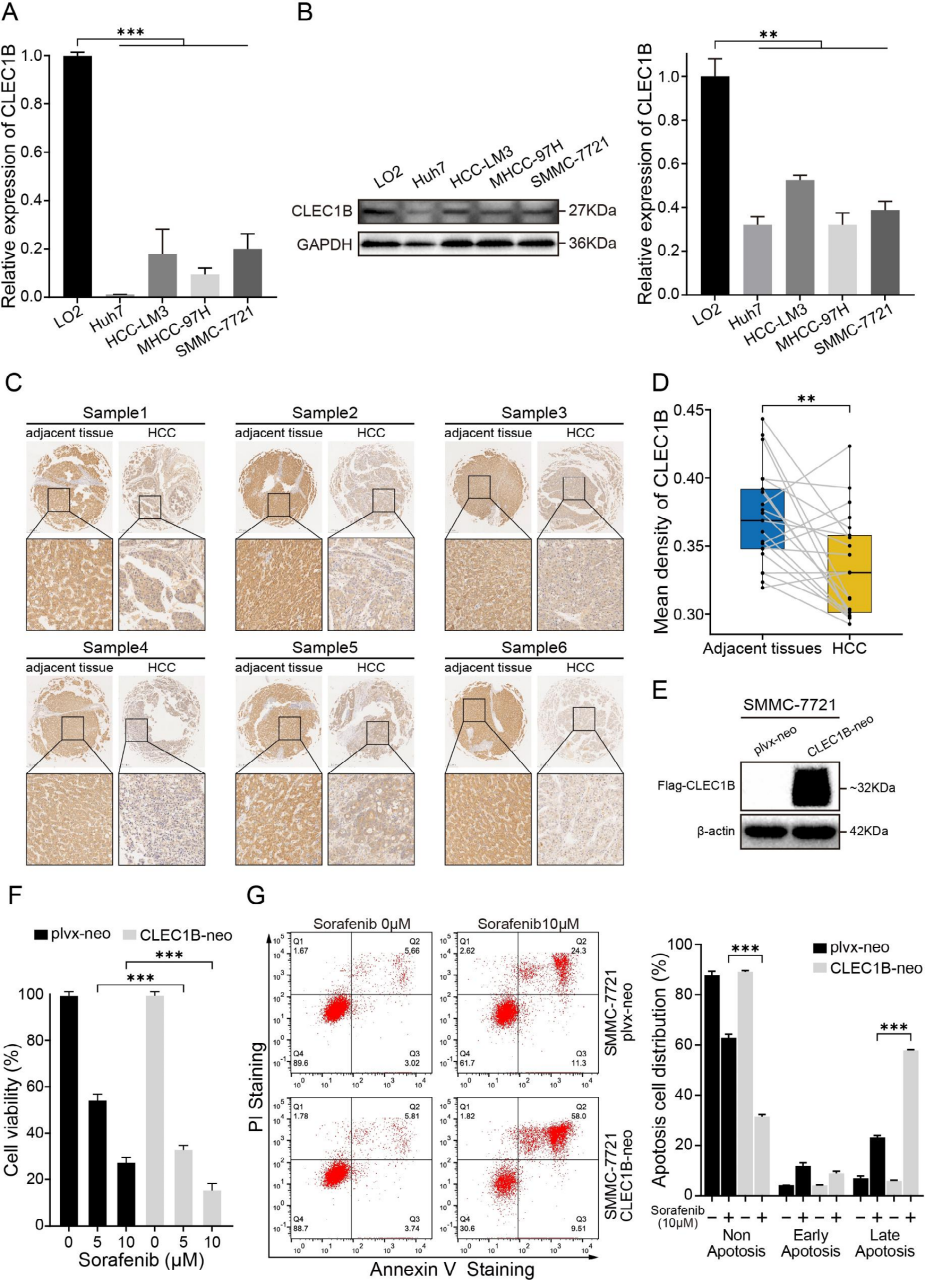
Expression validation of CLEC1B in HCC cells. (**A**, **B**) RT-qPCR (**A**) and western blot (**B**) detection of CLEC1B expression levels in HCC and normal hepatic liver cells. (**C**) Representative images of CLEC1B immunohistochemical staining of paired HCC and adjacent normal tissues. (**D**) Histogram shows the quantification data of immunohistochemical staining. (**E**) Protein expression of CLEC1B in SMMC-7721 plvx-neo and SMMC-7721 CLEC1B-neo cells. (**F**) Cell viability of SMMC-7721 plvx-neo and SMMC-7721 CLEC1B-neo upon sorafenib administration. (**G**) Flow cytometric analysis of apoptosis distribution after sorafenib treatment in SMMC-7721 cells, and the quantification data were shown on the right. **P-value < 0.01, ***P-value < 0.001

To confirm whether CLEC1B expression was lower in HCC than in normal hepatocytes, we performed RT-qPCR and western blot analysis. Lower mRNA and protein expression levels of CLEC1B were observed in human HCC cell lines (HCC-LM3, SMMC-7721, Huh7, and MHCC-97 H cells) compared with human normal hepatocyte LO2 cells (Fig. 8A, B). Meanwhile, immunohistochemical staining analysis underlines that CLC1B protein was down − regulated in HCC samples (Fig. 8C, D). To investigate whether the expression of CLEC1B exerted an impact on the sensitivity of the targeted drug, CLEC1B was overexpressed in SMMC-7721 cells. As shown in Fig. 8E, the protein level of CLEC1B was significantly overexpressed in SMMC-7721 cells. Cell viability analysis showed that sorafenib exhibited stronger cytotoxicity in SMMC-7721 CLEC1B-neo cells than in SMMC-7721 plvx-neo cells (Fig. 8F). Meanwhile, apoptosis analysis reflected that CLEC1B overexpressed SMMC-7721 cells were more sensitive to sorafenib (Fig. 8G), indicating that CLEC1B may be a potential target that can affect the cytotoxicity of sorafenib to HCC cells.

## Discussion

Most previous studies on CLEC1B have focused on its association with thrombosis, blood-lymphatic/vascular separation, and tumor metastasis [44–47]. The regulation and biological functions of CLEC1B in HCC remain largely undefined. In the current study, we found that CLEC1B, which is tightly bound to the immunological status of the TME, may be a robust biomarker for the diagnosis and prognosis of HCC. In addition, we discovered that CLEC1B significantly affects the cytotoxicity of sorafenib on HCC cells. Importantly, our study may provide insights into the deep exploration of the potential role of CLEC1B in HCC treatment.

We first assessed the transcriptional expression of CLEC1B in multiple cancer types, and the results showed a clear decrease in CLEC1B expression in most tumors, especially HCC [48]. The qPCR and western blot assays further validated the low mRNA and protein expression of CLEC1B in HCC cells. Immunohistochemical staining also confirmed the CLEC1B protein was down − regulated in HCC samples. Subcellular localization revealed that CLEC1B prevailed in the plasma membrane, which may have a potential role in signal transduction. Then, we explored the effect of CLEC1B on the survival outcomes of HCC patients, results proved that CLEC1B is a protective factor for HCC patients and that low expression is starkly associated with poor prognosis. The above results imply that CLEC1B may be a potential prognostic biomarker and plays a pivotal role in the tumorigenesis of HCC.

In recent years, immunotherapy, represented by immune checkpoint blockade and CAR-T cells, has changed the way conventional chemotherapy is undertaken. Although immunotherapy is an effective option for the treatment of tumors, it only benefits a minority of patients owing to the heterogeneity of tumors and the complicated TME of individual patients [49–51]. Therefore, it is imperative to identify potential biomarkers that can be used to predict the immunotherapy response. Our immune cells and single-cell analysis showed that CLEC1B is principally expressed in monocytes, PBMC, macrophages, granulocytes, myeloid DC, mast cells, and endothelial cells. We also found that CLEC1B had a highly positive correlation with the degree of immune infiltration of most infiltrating lymphocytes, including monocytes, Th1 cell, Th17 cell, Act B cell, Tem CD8, Act CD8, macrophage, neutrophil cell, eosinophil cell, NK cell, Treg cell, Tgd cell, and others; indicating that CLEC1B most likely participates in the development and survival status of HCC by changing the TME. Studies have revealed that CLEC-2 (coded by CLEC1B) is crucial for lymphatic cell proliferation and the maintenance of lymph nodes, and the migration of dendritic cells to lymph nodes depends on the DC-specific expression of CLEC-2 [52–54]. The activation of tumor-specific CD8 + T cells relies on the cross-presentation of APC antigens, such as DCs or macrophages [55, 56], suggesting that CLEC1B may affect the antigen cross-presentation of APCs and immune activation. These chemokines (CXCL9, CXCL10, CXCL11, CXCL13, CCL2, CCL3, CCL4, CCL5, and CCL21) and their receptors (CCR1, CCR2, CCR5, CCR6, and CXCR3) show a positive correlation with CLEC1B, indicating that CLEC1B plays a pivotal role in the recruitment of CD8 + T cells, TH17 cells, antigen-presenting cells, and other tumor-infiltrating immune cells [57]. In addition, the GSEA results further confirmed that CLEC1B was closely associated with the immune activation process, including the regulation of “allograft rejection”, “IFN α response”, “IFN γ response”, “inflammatory response”, and “TNFA signaling via NFKB pathways” [58]. These pathways have been proven to have non-negligible effects on immune infiltration, the immunotherapy response, and prognosis [59].

Further analysis shed light on the robust role of CLEC1B in the immune response, which is involved in the biological processes of immune cell activation, differentiation, and proliferation. The KEGG analysis of CLEC1B revealed a marked association with immune-related pathways such as the “cytokine-cytokine receptor interaction”, “chemokine signaling pathway”, and “T cell receptor signaling pathway”. Previous reports have confirmed that the expression of PI3K subunits is inhibited by CLEC2 in an SYK-dependent fashion, and CLEC2 elicits powerful platelet activation in combination with SYK [60–62]. CLEC2 is involved in the activation of the AKT/MAPK pathway in platelets via secondary mediators such as ADP and TxA2 [63, 64]. In addition to immune regulation, our study confirmed that CLEC1B is linked to platelet activation, the PI3K-Akt signaling pathway, and the MAPK signaling pathway.

Studies have shown that sorafenib, as one of the hepatoma-targeting drugs, exhibited advanced anti-tumor effects in HCC. Our results signaled that the expression of CLEC1B significantly affects the cytotoxicity of sorafenib to HCC cells. Despite these findings, several limitations are existing in our current study. The data of HCC samples were retrieved from public databases. It is, importantly, unclear why CLEC1B expression affects the therapeutic effect of sorafenib in HCC cells, the detailed mechanisms were not demonstrated. Therefore, further studies with more detailed molecular mechanisms involved were required.

## Conclusion

In conclusion, our comprehensive analysis of CLEC1B highlighted its potential effect on immune regulation and the prediction of HCC prognosis. We believe that CLEC1B is a promising prognostic biomarker for HCC.

## Electronic supplementary material

Below is the link to the electronic supplementary material.

**Additional File 1:** Figure S1. CLEC1B expression and distribution. (A) The expression of CLEC1B in normal human tissues. (B) Histogram of the intracellular distribution of CLEC1B. (C) Protein expression of CLEC1B in plasma as determined by mass spectrometry. (D) Scatter plot of CLEC1B expression in HCC normal and tumor samples. (E) CLEC1B expression in paired tumor tissues and paracancerous tissues of HCC. (F) ROC curve of CLEC1B in HCC. ***P-value < 0.001.

**Additional File 2:** Figure S2. CLEC1B expression in HCC and adjacent normal tissues. (A-D) the expression level of CLEC1B in cohorts of HCC patients is based on reports by Chen Liver (A), Roessler Liver 2 (B), Roessler Liver (C), and Wurmbach Liver (D). (E, F) CLEC1B expression in 11 HCC datasets based on the HCCDB database.

**Additional File 3:** Figure S3. Clinical significance of CLEC1B in HCC. (A-C) Effect of CLEC1B on OS, DSS, and PFI events in HCC. (D) Univariate Cox regression analysis of CLEC1B in HCC samples. The green stand for a protective factor. *P-value < 0.05.

**Additional File 4:** Figure S4. Heatmap shows the co-expression differences of 20 CLEC1B interacted proteins in HCC. *P-value < 0.05, ***P-value < 0.001.

**Additional File 5:** Supplementary Tables

## Data Availability

All data generated during this study are included either in this article or in the supplementary information files.
