# Supplementary figures and images for: Comprehensive analysis identifies CLEC1B as a potential prognostic biomarker in hepatocellular carcinoma
Source: Cancer Cell Int. 2023 Jun 12;23:113. doi: 10.1186/s12935-023-02939-1 (PMC10262401; doi:10.1186/s12935-023-02939-1)

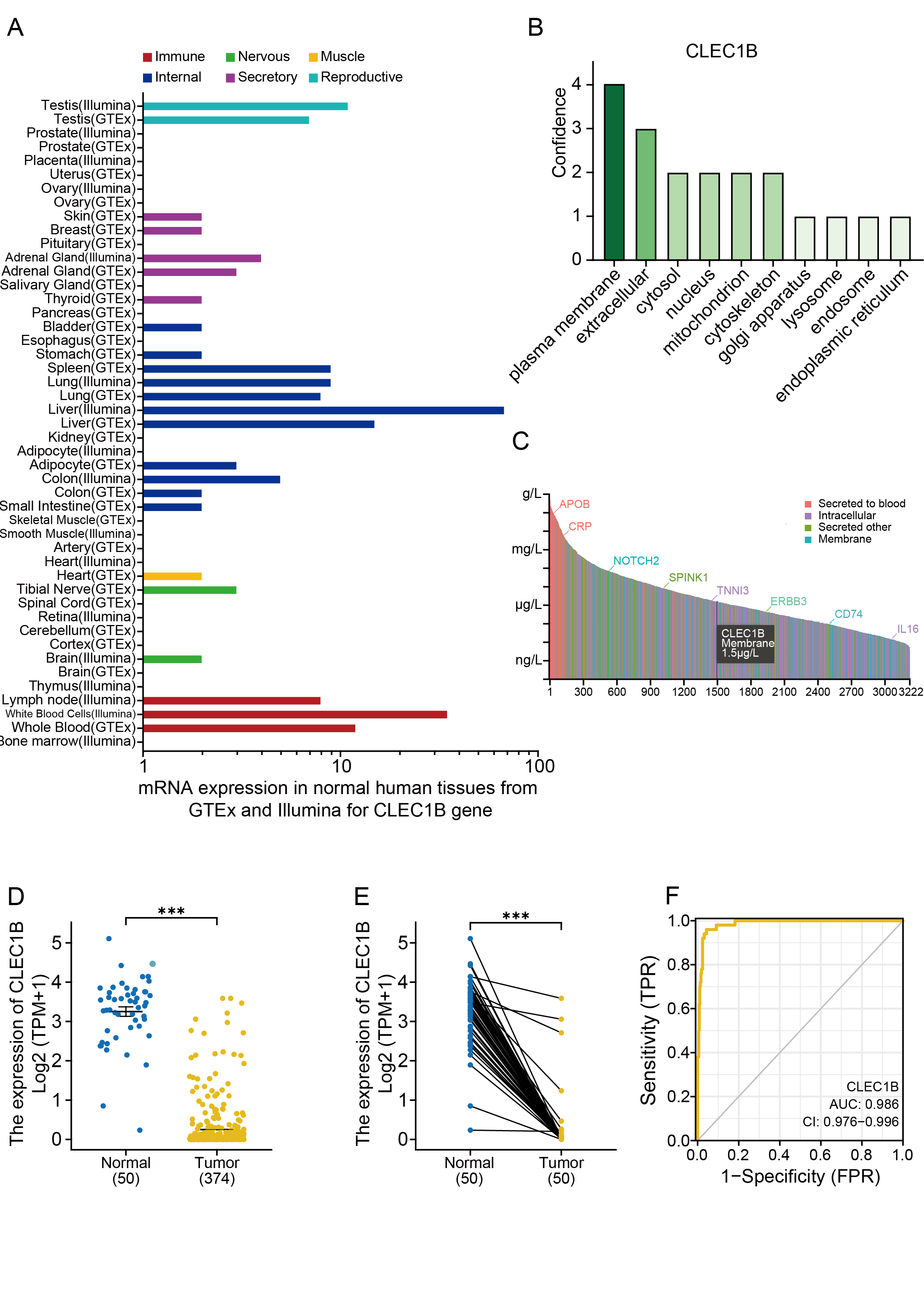

Supplement: Supplementary file 1 — Additional File 1: Figure S1. CLEC1B expression and distribution. (A) The expression of CLEC1B in normal human tissues. (B) Histogram of the intracellular distribution of CLEC1B. (C) Protein expression of CLEC1B in plasma as determined by mass spectrometry. (D) Scatter plot of CLEC1B expression in HCC normal and tumor samples. (E) CLEC1B expression in paired tumor tissues and paracancerous tissues of HCC. (F) ROC curve of CLEC1B in HCC. ***P-value < 0.001. [file 12935_2023_2939_MOESM1_ESM.jpg]

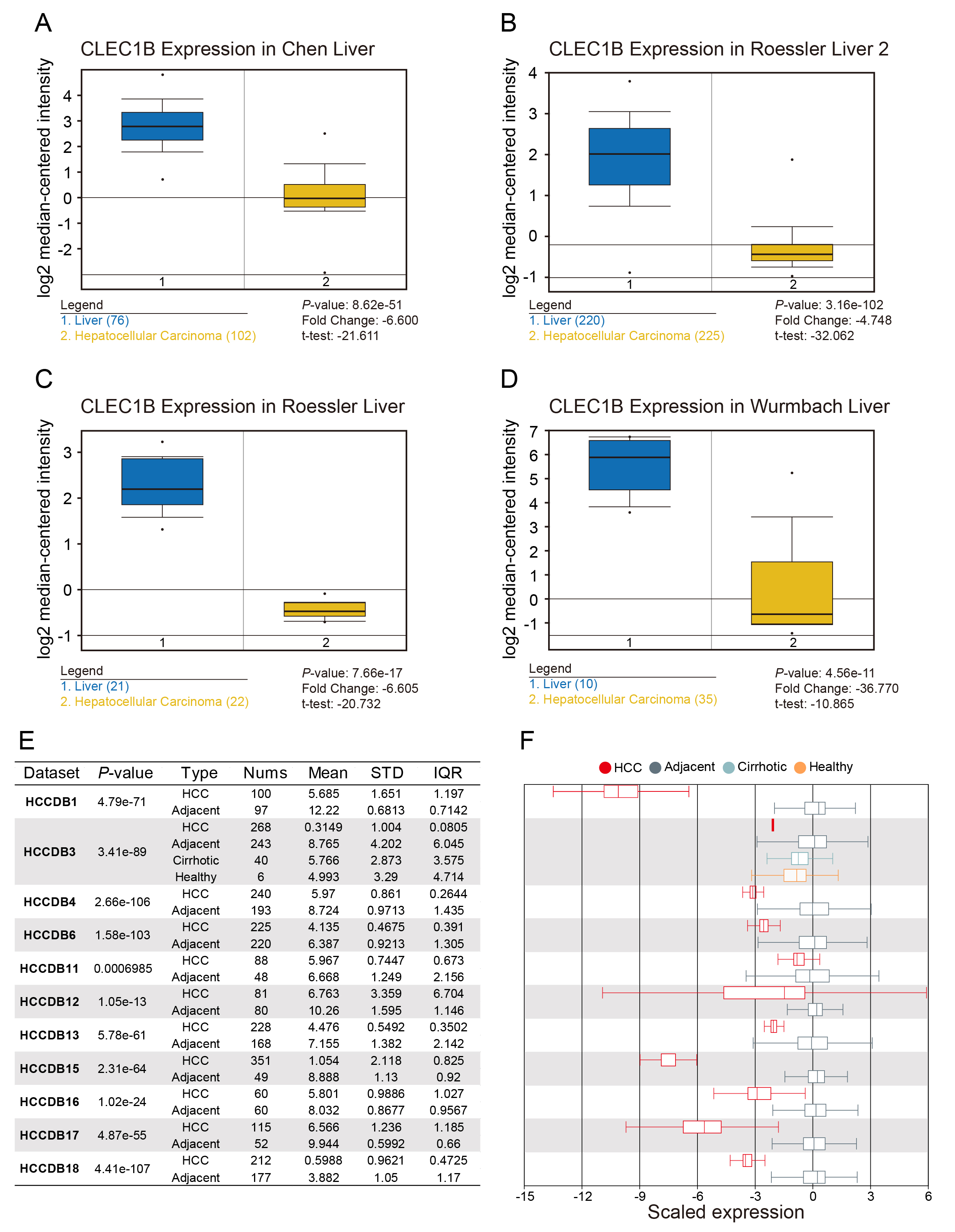

Supplement: Supplementary file 2 — Additional File 2: Figure S2. CLEC1B expression in HCC and adjacent normal tissues. (A-D) the expression level of CLEC1B in cohorts of HCC patients is based on reports by Chen Liver (A), Roessler Liver 2 (B), Roessler Liver (C), and Wurmbach Liver (D). (E, F) CLEC1B expression in 11 HCC datasets based on the HCCDB database. [file 12935_2023_2939_MOESM2_ESM.jpg]

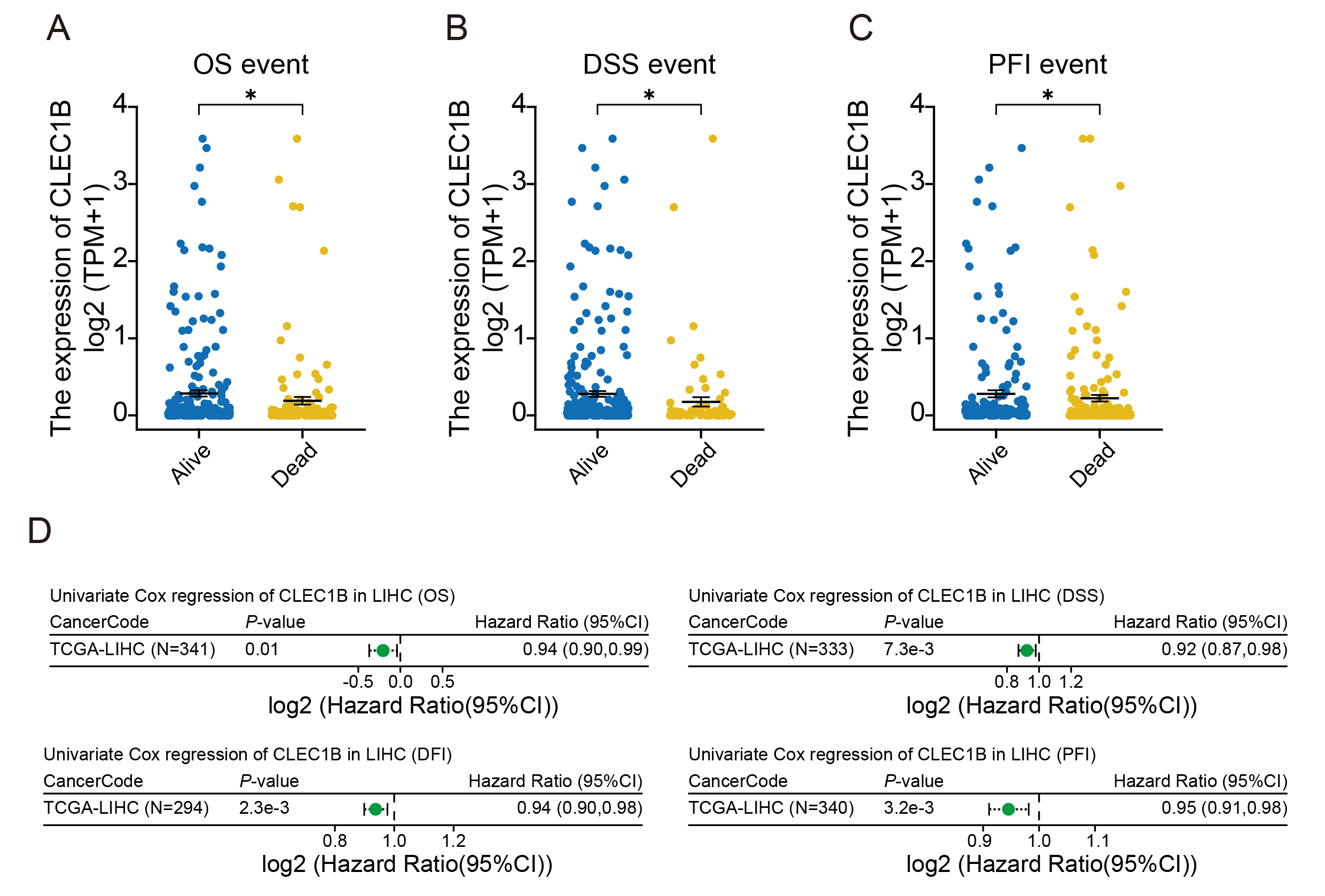

Supplement: Supplementary file 3 — Additional File 3: Figure S3. Clinical significance of CLEC1B in HCC. (A-C) Effect of CLEC1B on OS, DSS, and PFI events in HCC. (D) Univariate Cox regression analysis of CLEC1B in HCC samples. The green stand for a protective factor. *P-value < 0.05. [file 12935_2023_2939_MOESM3_ESM.jpg]

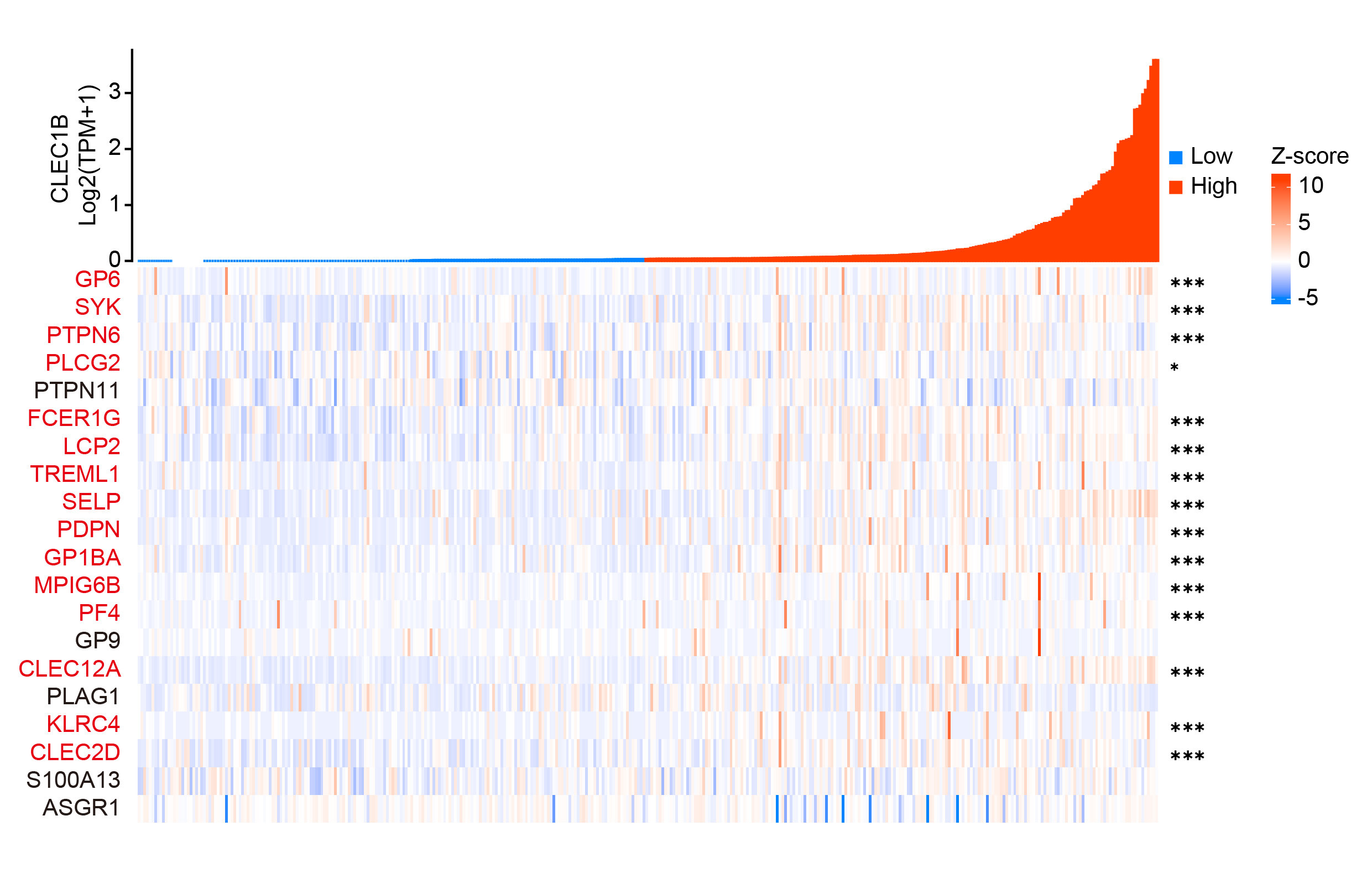

Supplement: Supplementary file 4 — Additional File 4: Figure S4. Heatmap shows the co-expression differences of 20 CLEC1B interacted proteins in HCC. *P-value < 0.05, ***P-value < 0.001. [file 12935_2023_2939_MOESM4_ESM.jpg]
