# Supplementary material for: Comprehensive analysis identifies CLEC1B as a potential prognostic biomarker in hepatocellular carcinoma
Source: Cancer Cell Int. 2023 Jun 12;23:113. doi: 10.1186/s12935-023-02939-1 (PMC10262401; doi:10.1186/s12935-023-02939-1)
Supplement: Supplementary file 5 — Additional File 5: Supplementary Tables [file 12935_2023_2939_MOESM5_ESM.docx]

Table S1. Characterization platforms used and data derived

| Data set | Cases | Data access |
| --- | --- | --- |
| GSE121248 | Normal (37), Tumor (70) | Open |
| GSE76427 | Normal (52), Tumor (115) | Open |
| GSE36376 | Normal (193), Tumor (240) | Open |
| GSE60502 | Normal (18), Tumor (18) | Open |

Table S2. Correlation between CLEC1B expression and the abundance of immune-related signatures of 28 TIL types in LIHC.

| Description | CLEC1B |  |
| --- | --- | --- |
|  | Purity |  |
|  | Correlation coefficient | *P*-value |
| Act CD8 | 0.182 | <0.001 |
| Tcm CD8 | 0.019 | 0.713 |
| Tem CD8 | 0.228 | <0.001 |
| Act CD4 | -0.17 | 0.001 |
| Tcm CD4 | 0.046 | 0.377 |
| Tem CD4 | -0.057 | 0.274 |
| Tfh | 0.108 | 0.0379 |
| Tgd | 0.125 | 0.016 |
| Th1 | 0.288 | <0.001 |
| Th17 | 0.117 | 0.0234 |
| Th2 | -0.053 | 0.307 |
| Treg | 0.163 | 0.00164 |
| Act B | 0.257 | <0.001 |
| Imm B | 0.119 | 0.0217 |
| Mem B | 0.022 | 0.667 |
| NK | 0.184 | <0.001 |
| CD56bright | 0.099 | 0.0567 |
| CD56dim | 0.119 | 0.0217 |
| MDSC | 0.17 | 0.00101 |
| NKT | 0.116 | 0.0253 |
| Act DC | 0.027 | 0.602 |
| pDC | 0.122 | 0.0181 |
| iDC | 0.086 | 0.0976 |
| Macrophage | 0.227 | <0.001 |
| Eosinophil | 0.206 | <0.001 |
| Mast | 0.234 | <0.001 |
| Monocyte | 0.296 | <0.001 |
| Neutrophil | 0.21 | <0.001 |

Table S3. Correlation of CLEC1B with other 20 genes

| Target gene | Other genes | Correlation coefficient | *P*-value |
| --- | --- | --- | --- |
| CLEC1B | CLEC12A | 0.543 | <0.001 |
|  | SELP | 0.442 | <0.001 |
|  | LCP2 | 0.424 | <0.001 |
|  | SYK | 0.369 | <0.001 |
|  | GP1BA | 0.337 | <0.001 |
|  | FCER1G | 0.328 | <0.001 |
|  | CLEC2D | 0.323 | <0.001 |
|  | PDPN | 0.315 | <0.001 |
|  | TREML1 | 0.261 | <0.001 |
|  | MPIG6B | 0.248 | <0.001 |
|  | GP6 | 0.240 | <0.001 |
|  | PF4 | 0.226 | <0.001 |
|  | PTPN6 | 0.196 | <0.001 |
|  | KLRC4 | 0.193 | <0.001 |
|  | PLCG2 | 0.113 | 0.030 |
|  | S100A13 | 0.093 | 0.072 |
|  | PTPN11 | 0.073 | 0.161 |
|  | PLAG1 | 0.042 | 0.417 |
|  | ASGR1 | -0.041 | 0.428 |
|  | GP9 | -0.029 | 0.571 |

**Supplementary figure legends**

Figure S1. CLEC1B expression and distribution. (A) The expression of CLEC1B in normal human tissues. (B) Histogram of the intracellular distribution of CLEC1B. (C) Protein expression of CLEC1B in plasma as determined by mass spectrometry. (D) Scatter plot of CLEC1B expression in HCC normal and tumor samples. (E) CLEC1B expression in paired tumor tissues and paracancerous tissues of HCC. (F) ROC curve of CLEC1B in HCC. ****P*−value < 0.001.

Figure S2. CLEC1B expression in HCC and adjacent normal tissues. (A−D) the expression level of CLEC1B in cohorts of HCC patients is based on reports by Chen Liver (A), Roessler Liver 2 (B), Roessler Liver (C), and Wurmbach Liver (D). (E, F) CLEC1B expression in 11 HCC datasets based on the HCCDB database.

Figure S3. Clinical significance of CLEC1B in HCC. (A−C) Effect of CLEC1B on OS, DSS, and PFI events in HCC. (D) Univariate Cox regression analysis of CLEC1B in HCC samples. The green stand for a protective factor. **P*−value < 0.05.

Figure S4. Heatmap shows the co−expression differences of 20 CLEC1B interacted proteins in HCC. **P*−value < 0.05, ****P*−value < 0.001.
